# *IFNAR2* Deficiency Causing Dysregulation of NK Cell Functions and Presenting With Hemophagocytic Lymphohistiocytosis

**DOI:** 10.3389/fgene.2020.00937

**Published:** 2020-09-18

**Authors:** Chiara Passarelli, Adele Civino, Marianna N. Rossi, Loredana Cifaldi, Valentina Lanari, Gian Marco Moneta, Ivan Caiello, Claudia Bracaglia, Raffaele Montinaro, Antonio Novelli, Fabrizio De Benedetti, Giusi Prencipe

**Affiliations:** ^1^U.O.C. Laboratory of Medical Genetics, Bambino Gesù Children’s Hospital, IRCCS, Rome, Italy; ^2^Rheumatology and Paediatric Immunology, PO “Vito Fazzi,” Lecce, Italy; ^3^Division of Rheumatology, Bambino Gesù Children’s Hospital, IRCCS, Rome, Italy; ^4^Academic Department of Pediatrics (DPUO), Bambino Gesù Children’s Hospital, IRCCS, Rome, Italy; ^5^Department of Clinical Sciences and Translational Medicine, University of Rome “Tor Vergata”, Rome, Italy; ^6^Division of Pediatrics, PO “Santa Caterina Novella,” Galatina, Italy

**Keywords:** hemophagocityc lymphohistiocytosis, interferon, IFNAR2, NK cells, clinical exome

## Abstract

We describe a 2 year old boy with two previously undescribed frameshift mutations in the interferon (IFN)α/β receptor 2 (*IFNAR2*) gene presenting with hemophagocytic lymphohistiocytosis (HLH) following measles-mumps-rubella vaccination. Functional analyses show the absence of response to type I IFN in the patient’s cells, as revealed by the lack of phosphorylation of STAT1 and the lack of induction of interferon-stimulated genes upon *ex vivo* stimulation with IFNα. HLH has been reported in patients with inborn errors of type I IFN-mediated immune responses following vaccination with live-attenuated viruses. The relation between HLH and defective type I IFN-mediated responses is unclear. We show that in patient’s natural killer (NK) cells stimulated with IFNα the expected increase in degranulation and inhibition of IFNγ production were affected. These data support a role for NK cell function dysregulation and lack of inhibition of IFNγ production as contributors to the development of HLH in patients with impaired type I IFN signaling.

## Introduction

Hemophagocityc lymphohistiocytosis (HLH) is a life-threatening syndrome characterized by a hyperinflammatory state, caused by an overwhelming activation of T lymphocytes and macrophages. HLH is typically caused by biallelic null mutations in genes encoding proteins involved in the cytotoxic activity of T lymphocytes and natural killer (NK) cells (primary HLH) (Canna and Marsh, 2020). HLH can also frequently occur, in the absence of known genetic causes, in the context of malignancies, infections and rheumatic diseases (referred to as secondary HLH) (Canna and Marsh, 2020). A large body of evidence demonstrates the pivotal role of interferon (IFN)-γ in the pathogenesis of both primary and secondary HLH forms (Jordan et al., 2004; Bracaglia et al., 2017). HLH has also been reported in patients with inherited primary immunodeficiencies, including those caused by inborn errors of type I IFN-mediated immune responses. Type I IFNs (IFN α and β) play a crucial role in anti-viral immunity. They mediate their activity by binding to the heterodimeric receptor, composed of IFN-α/β receptor 1 (IFNAR1) and IFN-α/β receptor 2 (IFNAR2), activating JAKs kinases and phosphorylating STAT1 and STAT2, which, in turn, lead to transcription of IFN-signature genes (ISG) (Platanias, 2005). Recently, patients with deficiency in *IFNAR1*, *IFNAR2*, *STAT1*, and *STAT2* genes developing HLH have been described (Duncan et al., 2015; Burns et al., 2016; Hoyos-Bachiloglu et al., 2017; Alosaimi et al., 2019; Boehmer et al., 2020). The exact molecular mechanism underlying the development of HLH in these patients remains to date unknown. In the majority of these patients, HLH developed following inoculation with live-attenuated virus vaccines or virus infection.

In this study we report an IFNAR2 deficient patient who, similarly to the previously reported only case of *IFNAR2* deficiency, also developed HLH. We demonstrated the absence of response to type I IFN in the patient’s cells and we investigated whether *IFNAR2* deficiency affected patient’s NK cell functions.

## Methods

### Patient and Controls

The study was approved by the Bambino Gesù Children’s Hospital Ethical committee. Written informed consent was obtained from the individual(s) and/or minor(s)’ legal guardian/next of kin for the publication of any potentially identifiable images or data included in this article.

### Genetics Analysis

DNA was extracted from peripheral blood with QIAgen columns (QIAsymphony DNA minikit, Qiagen, Hilden, Germany) according to the manufacturer’s instructions. Concentration and purity of DNA samples were quantified by ND-1000 spectrophotometer (NanoDrop; Thermo Scientific, Waltham, MA, United States) and by FLx800 Fluorescence Reader (BioTek, Winooski, VT, United States). Clinical Exome, using a custom panel including 6920 genes known as associated to genetic diseases, was performed on genomic DNA by using the SeqCap EZ Enrichment Kit (Roche) according to the manufacture’s protocol on a NovaSeq6000 platform (Illumina). The reads were aligned to human genome build GRCh37/UCSC hg19. The BWA Enrichment application of BaseSpace (Illumina) and the TGex software (LifeMap Sciences, Inc.) were used for the variant calling and annotating variants, respectively. Sequence data were carefully analyzed and the presence of all suspected variants was checked in the public databases (dbSNP, 1000 Genomes, Exome Aggregation Consortium (ExAC) and Genome Aggregation Database (gnomAD). Putative disease-associated sequence variants were distinguished from polymorphisms using the following filtering criteria: an allele frequency below 1% in ExAC, species conservation of the underlying amino acid and a change in the protein’s primary structure. The variants were evaluated by VarSome (Kopanos et al., 2019) and categorized in accordance with the ACMG recommendations (Richards et al., 2015). Variants were examined for coverage and Qscore (minimum threshold of 30) and visualized by the Integrative Genome Viewer (IGV). The IFNAR2 (NM_207585) p.Leu79Ter (rs1310889473) and p.Ile185MetfsTer12 (rs1312285586) variants were confirmed in the patient and segregation studies were performed in his parents and sister by Sanger sequencing, following a standard protocol (BigDye Terminator v3.1 Cycle Sequencing Kit, Applied Biosystems by Life Technologies). Both mutations are reported in the Exome Aggregation Consortium/Genome Aggregation Database (gnomAD) with very low allelic frequencies (0.000004064 and 0.00001600, respectively) and were predicted to be damaging by *in silico* tools.

### STAT1 Phosphorylation

Fresh whole blood cells were left unstimulated or stimulated with 10 ng/ml of recombinant IFNα2β (Intron A, Schering-Plough) or IFNγ (BD Pharmigen) for 10 min at 37°C. Anti-CD3 and anti-CD14 (all from Becton Dickinson) staining was performed for 20 min at 4°C. Cells where then fixed with Lyse/Fix Buffer 10 min at 37°C and further incubated for 10 min at RT with FcBlock (1:200) in Stain Buffer (all from Becton Dickinson). After permeabilization with Perm Buffer II (BD PhosFlow), samples were stained with antibodies against phosphorylated Tyrosine (701) STAT1 (pSTAT1) or isotype control antibody for 20 min at 4°C. Monocytes were gated based on CD14 expression; NK cells were gated based on CD3^–^CD56^dim^CD16^+^ subset. All antibodies were purchased from BD Biosciences. Samples were run on a BD LSRFortessa X−20 instrument (BD Biosciences) and data were analyzed with FlowJo software, version 8.3 (Tree Star).

### RNA Isolation and Quantitative Real-Time PCR

Whole blood cells from patient and his family members were unstimulated or stimulated for 3 h with 10ng/ml of recombinant IFNα or IFNγ at 37°C. Total RNA was extracted using Trizol Reagent (Ambion), and cDNAs were obtained using the Superscript Vilo kit (Invitrogen). Gene expression levels of type I (*IFI27, IFI44L, CXCL10, ISG15, RSAD2, SIGLEC1*) and type II IFN-induced genes (*CXCL9, CXCL10, IDO1*) were evaluated by quantitative polymerase chain reaction (qPCR) (ABI Prism 7900 HT sequence detection platform, Applied Biosystems) with Taqman Universal PCR Mastermix and Gene-expression Assays (Applied Biosystems). The results were normalized using *GAPDH* (Applied Biosystems) as endogenous control. Data were analyzed with the 2Δct method and are expressed as fold difference.

### Chemokine Measurements

PBMCs were stimulated for 3 h with 10 ng/ml of recombinant IFNα or IFNγ at 37°C. CXCL10 levels in conditioned media were measured by Enzyme-Linked Immunosorbent Assays (ELISAs) (DuoSet ELISA KIT, R&D Systems, Minneapolis, Minnesota), accordingly to manufacturer instructions.

### NK Cell Degranulation and IFNγ Production Assays

1 × 10^6^ PBMCs from patient, his mother and two healthy children were freshly isolated by lymphocyte separation Ficoll centrifugation (LiStarFish) and cultured for 24 h in a 96-well plate with 200 μl of complete RPMI 1640 medium, in the absence or presence of IFNα (10 ng/ml) or IFNα (10 ng/ml). NK cell degranulation and intracellular IFNγ production assays were performed by co-culturing cells with K562 target cells, at an effector: target cell ratio of 10:1, in RPMI 1640 medium supplemented with 10% of fetal calf serum at 37°C for 4 h in presence of anti-CD107a-APC. After 1 h, GolgiStop solution (BD PharMingen) was added to the co-culture. Cells were then stained with anti-CD56-PE-Cy7,anti-CD16-FITC and anti-CD3-Alexa Fluor 700 and anti-CD107a-APC antibodies to evaluate the NK cell degranulation by flow cytometry. In parallel, after surface staining, cells were fixed and permeabilized with Cytofix/Cytoperm buffer, stained with the anti-IFNγ-PE antibody and analyzed by flow cytometry. All antibodies were purchased from BD Biosciences.

## Results and Discussion

We describe a Caucasian boy, born to non-consanguineous parents, with two novel frameshift mutations in the *IFNAR2* gene, which codify for one of the two chains of the IFNα/β receptor, who developed HLH. At 22 months of age, 5 days after inoculation with the live-attenuated measles-mumps-rubella (MMR) vaccine, he was hospitalized with high fever and lethargy. Continuous high fever, unresponsive to antibiotic therapy, irritability, myoclonic movements, cervical lymphadenopathy and maculopapular rash characterized the clinical course. Lumbar puncture was negative and laboratory parameters were highly suggestive for HLH, with progressive decrease in cell blood count, hyperferritinemia (4008 μg/L), elevation of liver enzymes (AST 360 U/L, ALT 550 U/L), lactate dehydrogenase (3155 U/L) and triglycerides (338 mg/dl), and hypofibrinogenemia (92.2 mg/dl). He received intravenous methylprednisolone pulses (30 mg/kg/day for 3 consecutive days) followed by high dose glucocorticoids (2 mg/kg) with progressive improvement of clinical and laboratory features. When he was 3 years old, he was admitted for febrile seizure and rash with an increase in acute phase reactants, but no evidence of HLH. He recovered in a few days with antibiotic therapy. Three months later, he presented a new febrile episode characterized by cough, stomatitis, cervical lymphadenomegaly and right basal pneumonia. A combined infection by Influenza A and Herpes Simplex virus was documented and he gradually improved with antibiotic and antiviral treatment. The patient is now 4 years old, he is in good general condition and he is growing normally. After one dose of MMR vaccine, he shows protective IgG levels for measles and rubella.

The patient’s parents and sister, who carry heterozygous variant of *IFNAR2* gene, are in good clinical condition, without history of major infection or hyperinflammatory episodes, nor vaccination reactions.

**FIGURE 1:**
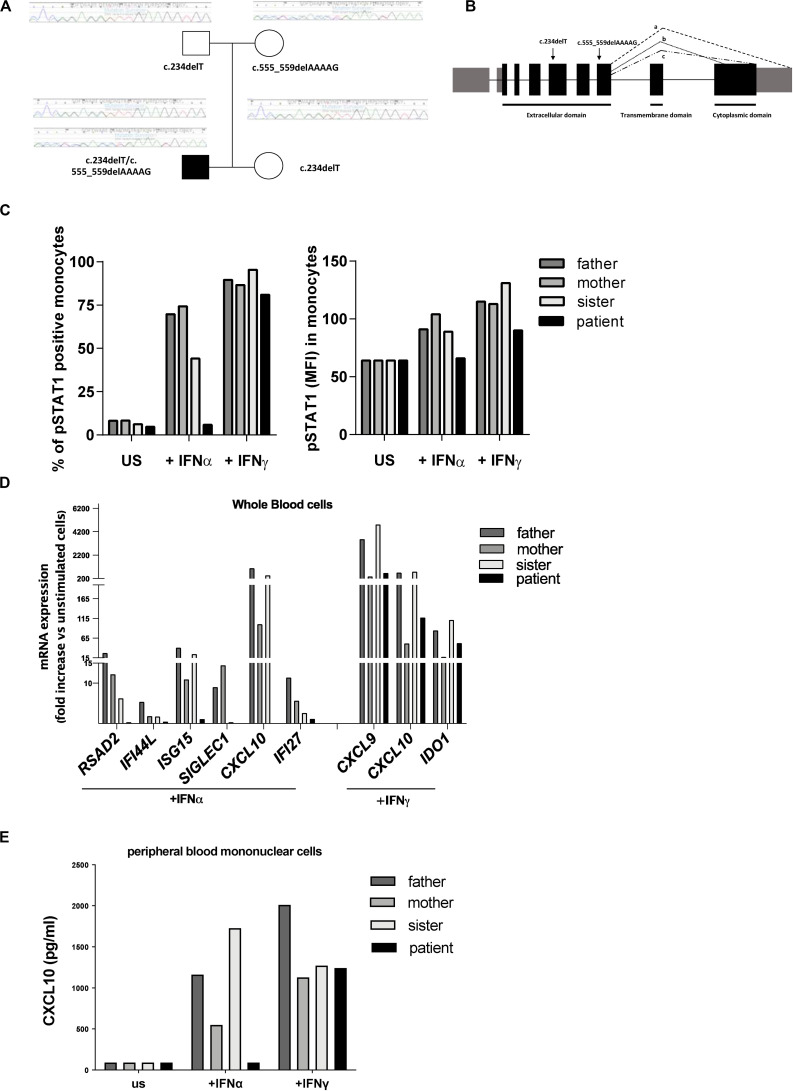
Genetic and functional findings in *IFNAR2* mutated patient. **(A)** Pedigree shows two novel frameshift mutations in *IFNAR2* gene, c.234delT and c.555_559delAAAAG, resulting, respectively, in p.Leu79Ter and p.Ile185MetfsTer12 variants. **(B)** Both variants are predicted to truncate the three known IFNAR2 isoforms (a–c). **(C)** Percentage of Tyrosine 701-phosphorylated STAT1 (pSTAT1) positive monocytes and mean of fluorescence intensity (MFI) of pSTAT1 in monocytes were assessed by flow cytometric analysis, following *ex vivo* incubation of whole blood cells with medium (unstimulated, US) in the absence or in the presence of IFNα (10 ng/ml) or IFNγ (10 ng/ml). **(D)** mRNA expression of type I IFN genes (*RSAD2, IFI44, ISG15, SIGLEC1, CXCL10, IFI27*) and type II IFN-regulated genes (*CXCL9, CXCL10, IDO1*) in IFNα and IFNγ *ex vivo* stimulated whole blood cells. Results were normalized with the housekeeping gene *GAPDH* and expressed as fold increase compared to unstimulated cells. **(E)** CXCL10 protein levels in supernatant of peripheral blood mononuclear cells unstimulated or stimulated for 3 h with IFNα or IFNγ were measured by ELISA.

Clinical exome analysis revealed two previously undescribed frameshift mutations in *IFNAR2* gene, c.234delT and c.555_559delAAAAG, resulting respectively in p.Leu79Ter and p.Ile185MetfsTer12 changes. The mutations were in a compound heterozygous status, as revealed by segregation studies on the parents; the asymptomatic sister carried the c.234delT mutation (Figure 1A). Both mutations were predicted to be damaging by *in silico* tools, since they introduce premature stop codons leading to the putative complete lack of the protein (Figure 1B). No additional mutations in genes associated with familial HLH were identified.

To investigate whether the predicted *IFNAR2* deficiency affected responses to type I IFN, whole blood cells from the patient and other family members were stimulated *ex vivo* with IFNα and the phosphorylation of STAT1 was evaluated. Absence of phosphorylated STAT1 in patient’s monocytes stimulated with IFNα was observed (Figure 1C). Accordingly, stimulation of whole blood cells with IFNα did not induce phosphorylation of STAT1 even in patient’s NK cells (data not shown). In the *IFNAR2* mutated patient, *ex vivo* stimulation of whole blood cells with IFNα failed to activate the transcription of type I ISG (Figure 1D). As expected, IFNγ stimulation was able to induce both STAT1 phosphorylation and transcription of type II IFN-regulated genes (Figures 1C,D). Consistently with the mRNA expression data, IFNα stimulation of peripheral blood mononuclear cells (PBMCs) isolated from the patient did not induce the production of CXCL10, while stimulation with IFNγ induced CXCL10 release in amounts similar to those observed in other family members (Figure 1E). Altogether, these results showed that in the patient’s immune cells the IFNα-mediated response was abolished.

Similarly to the previously reported only case of *IFNAR2* deficiency and to other patients with deficiency in *IFNAR1*, *STAT1*, and *STAT2* (Duncan et al., 2015; Burns et al., 2016; Hoyos-Bachiloglu et al., 2017; Alosaimi et al., 2019; Boehmer et al., 2020), our patient also developed HLH following inoculation with live-attenuated virus vaccines. To date, the relation between HLH and defective type I IFN-mediated responses is unclear.

**FIGURE 2:**
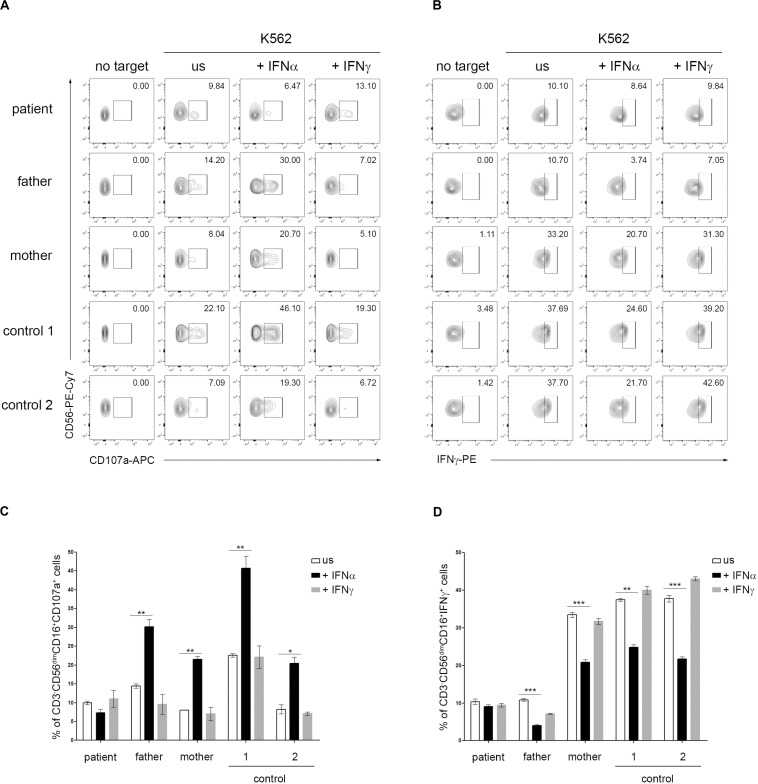
Dysregulation of natural killer (NK) cell functions in *IFNAR2* deficient patient. **(A–D)** PBMCs isolated from the patient, his parents and two pediatric controls were pretreated or not (unstimulated, US) for 16 h with 10 ng/ml of IFNα or IFNγ (as control) and then co-cultured with K562 target cells for 4 h, at an effector: target ratio of 10:1. **(A)** Representative dot plots of degranulation assay showing the expression of CD107a in CD3-CD56dim CD16 + NK cell subset. **(B)** Representative dot plots of intracellular IFNγ expression in CD3-CD56dim CD16 + NK cell subset. **(C,D)**, Percentage (mean ± *SD*, measured in triplicate) of CD107a^+^ and IFNγ^+^ cells in CD3^–^ CD56^dim^CD16^+^ cells subset were assessed by flow cytometric analysis (the *p*-value was calculated by two-tailed unpaired Student’s *t*-test, *p < 0.05, **p > 0.01, ***p < 0.001). Similar results were obtained in two independent experiments.

Type I IFNs exert their anti-viral effects by modulating the functions of both innate and adaptive immune cells. Several studies demonstrated a role for type I IFN receptor signaling in the activation of NK cell functions in response to viral infections (Paolini et al., 2015). In addition, a direct action of type I IFN on NK cells has also been demonstrated to be necessary for the innate immune defense against vaccinia viral infections (Martinez et al., 2008). A constitutive or transient defect in NK cell cytotoxicity typically occurs in patients with, respectively, primary and secondary HLH and is believed to play a key role in the pathogenesis of the disease (Grom, 2004; Janka, 2012; Vandenhaute et al., 2020). In order to evaluate whether *IFNAR2* deficiency affected NK cell functions, PBMCs isolated from the patient, his parents and two pediatric controls were pre-incubated with medium in the presence or in the absence of IFNα and CD107A expression, a marker for NK cell degranulation, was evaluated in response to exposure to K562 cells as target cells. Compared to control cells, we did not observe differences in NK cell degranulation in unstimulated patient cells. Interestingly, while pre-incubation of PBMCs with IFNα caused a marked increase (about 3-fold) in the percentage of NK cells expressing CD107a in the patient’s parents and controls, no increase was observed in NK cells of the patient (Figures 2A,C). These results are consistent with the previously demonstrated direct role of IFNα in inducing NK cell cytotoxic activity through IFNAR signaling (Paolini et al., 2015) and support a role for the impairment of NK cell functions in the development of HLH in our patient. Recent data demonstrate that type I IFN receptor negatively regulates IFNγ production in NK cells (Lee et al., 2019). We therefore evaluated the NK cell IFNγ production following incubation with IFNα. Consistently, we found that in patient’s NK cells IFNα treatment failed to reduce IFNγ production, compared to patient’s parents and control cells that, as expected, showed an evident decrease in intracellular IFNγ levels (Figures 2B,D).

Although these data need to be confirmed in a larger number of patients, they led us to speculate that in patients with IFNAR deficiency the attenuated type I IFN response to vaccinia viral infection, associated with dysregulation in NK cell functions (i.e., decreased NK cell cytotoxic activity and lack of inhibition of IFNγ production after exposure to type I IFN), contributes to the dissemination of vaccine viral infection and, also, to the development of HLH. This hypothesis may apply as well to patients with other inborn mutations affecting responses to type I IFN.

Our data in a patient with *IFNAR2* deficiency showing that impairment of type I IFN-mediated responses causes defective NK cell degranulation and lack of inhibition of IFNγ production further points to common mechanisms underlying the pathogenesis of primary and secondary HLH. From a clinical perspective, HLH episodes following administration of live-attenuated viral vaccine should be considered as suggestive of a defect in the type I IFN response. In these cases, as well as in children with recurrent viral infections with an aggressive and atypical course, rapid evaluation of phosphorylation of STAT1 and STAT2 by flow cytometry upon *ex vivo* stimulation with IFNα can guide clinicians in the early identification of patients with impairment in type I IFN-mediated signaling.

## Data Availability Statement

The datasets presented in this study can be found in online repository (ClinVar: https://www.ncbi.nlm.nih.gov/clinvar/). The accession numbers (rs number) can be found in the article.

## Ethics Statement

Written informed consent was obtained from the individual(s) and/or minor(s)’ legal guardian/next of kin for the publication of any potentially identifiable images or data included in this article.

## Author Contributions

GP, CP, and FD conceived and designed the work. CP, MR, LC, VL, GM, and IC performed the experiments. AC, RM, AN, and CB enrolled patient and controls. CP, LC, FD, and GP wrote the manuscript. All authors read and approved the final manuscript.

## Conflict of Interest

The authors declare that the research was conducted in the absence of any commercial or financial relationships that could be construed as a potential conflict of interest.

## Abbreviations

HLH: Hemophagocityc lymphohistiocytosis
IFN: Interferon
IFNAR1: IFN α / β receptor 1
IFNAR2: IFN α / β receptor 2
ISG: IFN-signature genes
MMR: measles-mumps-rubella
NK: Natural Killer
PBMCs: peripheral blood mononuclear cells.
